# Neural Entrainment and Sensorimotor Synchronization to the Beat in Children with Developmental Dyslexia: An EEG Study

**DOI:** 10.3389/fnins.2017.00360

**Published:** 2017-07-12

**Authors:** Lincoln J. Colling, Hannah L. Noble, Usha Goswami

**Affiliations:** Department of Psychology, Centre for Neuroscience in Education, University of Cambridge Cambridge, United Kingdom

**Keywords:** dyslexia, entrainment, synchronization, developmental disabilities, EEG, sensorimotor

## Abstract

Tapping in time to a metronome beat (hereafter beat synchronization) shows considerable variability in child populations, and individual differences in beat synchronization are reliably related to reading development. Children with developmental dyslexia show impairments in beat synchronization. These impairments may reflect deficiencies in auditory perception of the beat which in turn affect auditory-motor mapping, or may reflect an independent motor deficit. Here, we used a new methodology in EEG based on measuring beat-related steady-state evoked potentials (SS-EPs, Nozaradan et al., 2015) in an attempt to disentangle neural sensory and motor contributions to behavioral beat synchronization in children with dyslexia. Children tapped with both their left and right hands to every second beat of a metronome pulse delivered at 2.4 Hz, or listened passively to the beat. Analyses of preferred phase in EEG showed that the children with dyslexia had a significantly different preferred phase compared to control children in all conditions. Regarding SS-EPs, the groups differed significantly for the passive Auditory listening condition at 2.4 Hz, and showed a trend toward a difference in the Right hand tapping condition at 3.6 Hz (sensorimotor integration measure). The data suggest that neural rhythmic entrainment is atypical in children with dyslexia for both an auditory beat and during sensorimotor coupling (tapping). The data are relevant to a growing literature suggesting that rhythm-based interventions may help language processing in children with developmental disorders of language learning.

## Introduction

Sensorimotor synchronization refers to the rhythmic co-ordination of perception and action (Repp, 2005; Repp and Su, 2013), and is most often studied using finger tapping to a rhythmic sequence of auditory stimuli. Such rhythmic tapping is hereafter referred to as beat synchronization. The temporal co-ordination of rhythmic movement with an external rhythm tends to improve with age (e.g., Drake et al., 2000; McAuley et al., 2006), and is reliably enhanced with musical training (e.g., Thompson et al., 2015). The precision of beat synchronization is reduced in children with developmental dyslexia (Overy et al., 2003; Thomson and Goswami, 2008; Flaugnacco et al., 2014). In studies of child populations, the precision of beat synchronization is related to individual differences in both phonological awareness (children's ability to reflect on the sound structure of words, a cross-language predictor of the efficiency of reading acquisition) and to individual differences in progress in reading (e.g., Dellatolas et al., 2009 [French]; Corriveau and Goswami, 2009 [English]; Flaugnacco et al., 2014 [Italian]). Individual differences in beat synchronization are also related to measures of reading readiness (Woodruff Carr et al., 2014, English).

The mechanisms underlying the relationships between individual differences in beat synchronization, reading, and phonological awareness in children are currently unclear. To explore these relationships in more detail, here we adapt an EEG paradigm developed by Nozaradan and her colleagues for measuring selective neuronal entrainment to beat and meter based on steady-state evoked-potentials (SS-EPs, see Nozaradan et al., 2012, 2015). Nozaradan and colleagues were able to measure the emergence of an internal (neural) representation of beat at beat and meter frequencies that were not physically present in the stimulus. For example, Nozaradan et al. (2015) recorded EEG while adult participants listened to a 2.4 Hz metronome beat and tapped on every second beat (1.2 Hz). Neurally, both a 1.2 Hz SS-EP related to motor entrainment to the beat and a 2.4 Hz SS-EP related to auditory beat-related entrainment were observed, as would be expected. Crucially, an interaction between the sensory (2.4 Hz) and motor (1.2 Hz) SS-EPs was evidenced by an additional SS-EP at 3.6 Hz, compatible with sensorimotor integration and the internal representation of a beat. This approach offers a way of separating the sensory (auditory) and motor (manual) components of beat synchronization in child populations. Our question in the current study was whether children with developmental dyslexia, who are known to show deficits in both perceiving auditory rhythm (e.g., Huss et al., 2011) and in tapping tasks (e.g., Thomson and Goswami, 2008) would show neural differences compared to typically-developing children in either the motor (1.2 Hz SS-EP) or the auditory (2.4 Hz SS-EP) domain, or in both domains. A group difference for the 3.6 Hz SS-EP would suggest that sensorimotor integration and the internal representation of the beat is impaired in developmental dyslexia.

When neural measures are used to index developmental mechanisms that may underlie impaired performance by children with developmental disorders, it is important that the groups being compared are matched on the behavior under study (Shaywitz et al., 2007; Olulade et al., 2013). For example, phonological processing is known to be impaired in children with dyslexia, so to measure atypical neural processing during a phonological task, the children with dyslexia must be matched in performance to the typically-developing children in the task being studied (usually achieved via a reading-level match group design, see Olulade et al., 2013). In the current study, matching the children with dyslexia for tapping behavior to the typically-developing controls permits the inference that group differences in beat-related neural activity reflect developmental differences rather than differences in sensorimotor expertise. Accordingly, in the current study we took advantage of an ongoing training study in our laboratory in which children with developmental dyslexia received practice in tapping to a 2 Hz beat on a weekly basis (as part of a larger battery of phonological remediation activities). The children who volunteered for EEG were matched in their behavioral tapping performance to the age-matched control children, and both groups were then compared in Nozaradan et al.'s (2015) task. On the basis of our prior data (Power et al., 2013), we expected that neural differences in preferred phase in beat-related auditory entrainment would drive any group differences that we might observe. As our prior EEG studies revealed auditory neural differences between children with dyslexia and control children in the time domain rather than in the frequency domain, no *a priori* prediction was made regarding whether group differences may be found in the SS-EPs.

For children with developmental dyslexia, it is unknown whether their impairments in beat synchronization arise from a primary sensory deficit in auditory rhythm perception which in turn affects the temporal precision of action, or whether beat synchronization is impaired via an independent deficit related to the developing motor system, or both. As impairments in both rhythm perception and the precision of beat synchronization are found in a range of developmental disorders of language, including stuttering (Falk et al., 2015; Wieland et al., 2015) and speech and language impairment (Corriveau and Goswami, 2009; Cumming et al., 2015) as well as developmental dyslexia, it is of theoretical interest to ascertain the sensory and/or motor sources of impaired sensorimotor synchronization in children with different disorders. The identification of underlying neural mechanisms would help to optimize remediation. Synchronizing to a beat is a complex process, requiring accurate internal time-keeping, the production of steady movements, and the use of auditory feedback to predict and correct the timing of action (Sowinski and Dalla Bella, 2013; Cason et al., 2015; Tierney and Kraus, 2016). To take the example of dyslexia, if the primary sensory impairment is auditory rather than motor, then rhythmic interventions involving a motor component (e.g., drumming) may improve rhythmic synchronization in affected children via sensorimotor coupling, with consequent effects on auditory and language processing driven by the motor practice (Goswami, 2012). Linguistic improvements are indeed found for rhythmic interventions with children that involve drumming, or other forms of rhythm production (tapping with a pencil), or playing a musical instrument (e.g., Degé and Schwarzer, 2011; Bhide et al., 2013; Slater et al., 2014; Flaugnacco et al., 2015; Serrallach et al., 2016). For example, in Bhide et al. (2013), the rhythmic intervention (based on bongo drumming) improved children's discrimination of amplitude envelope “rise time,” phonological awareness and reading. Bhide et al. (2013) interpreted this cross-domain (motor-to-language) enhancement in terms of Temporal Sampling theory (Goswami, 2011).

Temporal Sampling (TS) theory has been developed as a sensory/neural explanatory framework for the linguistic (phonological) deficits that characterize children with developmental dyslexia across languages. TS theory proposes that phonological difficulties in developmental disorders of language may be related to atypical neural entrainment by auditory oscillatory networks to the rhythms of speech (the syllable “beats”) that are carried by slower amplitude modulations (AMs) <10 Hz. This atypical neural entrainment is thought to be related to impaired sensory discrimination of amplitude envelope (AE) rise times. Impaired discrimination of AE rise times is found in children with developmental dyslexia in a range of languages (English, French, Dutch, Spanish, Chinese, Hungarian, and Finnish, see Goswami, 2015, for a summary). Amplitude envelope rise time is an important perceptual correlate of rhythm. The onsets of successive modulations in the amplitude envelope (AE) of a sound, and the rates of change of these modulation onsets (AE rise times), are a key temporal component of rhythmic perceptual structure for both speech and music (e.g., Greenberg et al., 2003; Patel, 2010, 2012; Goswami, 2011; Giraud and Poeppel, 2012). As well as showing consistent relations with phonological awareness, the impairments in AE rise time discrimination in dyslexic populations are also usually linked behaviorally to impaired *non-speech* rhythmic processing, for example as measured by musical tasks requiring sensitivity to metric structure (see Huss et al., 2011; Goswami et al., 2013; Flaugnacco et al., 2015).

By TS theory, these well-documented behavioral relationships between AE rise time discrimination, rhythm discrimination, and phonological development are linked to atypical oscillatory neuronal entrainment to the AM patterns in speech and music that carry rhythm. Neural studies in adult populations have shown that speech is encoded in part via the entrainment of neuronal oscillations in auditory cortex by AMs in the speech signal at multiple temporal rates simultaneously [delta (~1–3 Hz), theta (~4–8 Hz), beta (~15–30 Hz), and gamma (>30 Hz); see (Giraud and Poeppel, 2012; Poeppel, 2014)]. These oscillating neural networks phase-reset their activity to phase-align or entrain with AMs in the speech signal, using AE rise times as acoustic landmarks for accurate alignment (see Gross et al., 2013; Doelling et al., 2014). As children with developmental dyslexia show impaired discrimination of AE rise times, their phase resetting process is likely to be atypical. Indeed, Power et al. (2013) demonstrated a *different preferred phase* in delta-band entrainment (~1–3 Hz) in children with dyslexia aged on average 13 years compared to control children in a rhythmic speech listening task (to syllable repetition at 2 Hz). Power et al.'s data suggested neuronal phase alignment to *less informative temporal points* in the incoming speech signal by children with dyslexia, which would affect the extraction of phonological information.

Interestingly, studies of beat synchronization in children reveal that 2 Hz (500 ms) is the preferred spontaneous tempo (the rate at which children choose to tap in the absence of an external timekeeper, see McAuley et al., 2006, see also Moelants, 2002). Accordingly, by TS theory, the impaired synchronization to the beat found in children with developmental dyslexia may arise from atypical auditory phase entrainment to acoustic rhythm in the delta band, which in turn affects sensorimotor synchronization. Related studies reveal that children with developmental dyslexia also show atypical neural entrainment to continuous speech in the delta band (centered on ~2 Hz, Power et al., 2016, sentence repetition, English; Molinaro et al., 2016, story comprehension, Spanish).

A complementary theoretical framework that is relevant to the development of accurate beat synchronization, but based on the mechanisms governing attention rather than linguistic processing, is Dynamic Attending Theory (DAT; Large and Jones, 1999; Jones, 2008). DAT proposes that better sensory processing should be found when the brain can utilize a regular rhythm for temporal prediction. By DAT, if an auditory rhythm is present, such as a 2.4 Hz beat train, then this can be thought of as an external oscillator at 2.4 Hz, with which hypothetical internal oscillators (neural rhythms with similar phase and period relations) can synchronize, thereby coupling their neural activity with the external oscillator. By DAT, successful rhythmic entrainment would dynamically modify attention in time, with more attentional resources being allocated to informative positions, such as the temporal point at which the beat occurred. As an example, a network of neurons whose preferred oscillatory rate was 2.4 Hz (the oscillations reflect the concentration of neuronal electrical discharges to particular phases of a temporal cycle) would entrain to an external auditory rhythm at 2.4 Hz, so that maximal neural activity (peak phase) coincided with the beat. Perception of other sensory information (e.g., a flash of light) that occurred during this enhanced attentional phase would then be optimized. Neural networks that oscillate quasi-rhythmically at different temporal rates are found in many areas of the brain (e.g., Buzsáki, 2004; Lakatos et al., 2008; Schroeder and Lakatos, 2009). It has further been demonstrated that these oscillators indeed affect attention. For example, adults are unaware of visual stimuli that occur during the trough (least excitable phase) of a parietal alpha oscillation (~10 Hz) and are most likely to detect targets at the oscillatory peak (Mathewson et al., 2009).

Although DAT focuses on attention and TS theory focuses on linguistic processing, both theoretical frameworks predict that children who can entrain successfully to an acoustic rhythm will be more likely to tap in time in beat synchronization tasks. These two theoretical frameworks used in the child beat synchronization literature are largely complementary, and enable some neural predictions to be made for the current study. Firstly, on TS theory, group differences in the *phase of entrainment* in the delta band (here, 2.4 Hz) would be expected in the time domain. Following Power et al. (2013), the children with dyslexia would be expected to show a difference in preferred phase angle compared to the typically-developing control children. Secondly, both DAT and TS theory would predict group differences in the internal representation of the beat, documented by the 3.6 Hz SS-EPs. Such a group difference would be indicative of disrupted sensorimotor integration in developmental dyslexia. Identification of the source/s of disrupted beat synchronization have important implications for optimizing educational remediation.

## Materials and methods

### Participants

Twenty-four children participated in the study, of whom 11 had a statement of developmental dyslexia from their local education authority and/or showed severe literacy and phonological deficits according to our own test battery. The participants were drawn from a cohort of children participating in a study testing the efficacy of behavioral interventions for developmental dyslexia, and comprised all those in the cohort consenting to EEG. The 11 children with dyslexia (DYS; 7 female, 4 male) and the 13 typically-developing children (CA; 6 female, 7 male) were matched for chronological age, as shown in Table 1. All children were right-handed. All participants and their guardians gave informed consent for EEG in accordance with the Declaration of Helsinki, and the study was approved by the Psychology Research Ethics Committee of the University of Cambridge. All participants were free of any diagnosed learning difficulties aside from dyslexia (i.e., dyspraxia, ADHD, autistic spectrum disorder, speech, and language impairments) and spoke English as their first language. Parental informed written consent was obtained for all participants, and all children had previously received a short hearing screen using an audiometer. Sounds were presented in both the left or right ear at a range of frequencies (250, 500, 1,000, 2,000, 4,000, 8,000 Hz), and all participants were sensitive to sounds within the 20 dB HL range.

**Table 1:** Participant information.

	**DYS**	**CA**	***t*-Values**	***P*-values**
Age in months	119.3 (11.3)	120.9 (7.9)	0.4	0.69
Reading age in months	83.0 (8.9)	109.9 (16.9)	5.0	<0.001
FSIQ	98.3 (7.6)	99.5 (9.2)	0.3	0.73
BAS reading SS	78.3 (4.7)	95.9 (8.8)	6.2	<0.001
BAS spelling SS	79.9 (6.9)	96.0 (10.7)	4.4	<0.001
TOWRE total SS	74.2 (8.8)	96.5 (14.9)	4.5	0.001
PhAB rhyme SS	82.9 (9.5)	103.3 (11.0)	4.9	<0.001
PhAB Rhyme #trials correct	9.3 (3.6)	17.5 (3.5)	5.6	<0.001
Rise time threshold 1 in ms	85.9 (54.2)	65.2 (37.0)	1.1	0.29
Rise time threshold 2 in ms	111.4 (74.6)	73.3 (55.1)	1.4	0.18
Duration threshold in ms	87.6 (21.1)	21.1 (23.8)	4.0	0.001

*DYS, Dyslexic, CA, Chronological Age control, FSIQ, Full Scale IQ, BAS, British Ability Scales, SS, standard score, TOWRE, Test of Word Reading Efficiency, PhAB, Phonological Assessment Battery*.

### Stimuli

**Figure 1:**
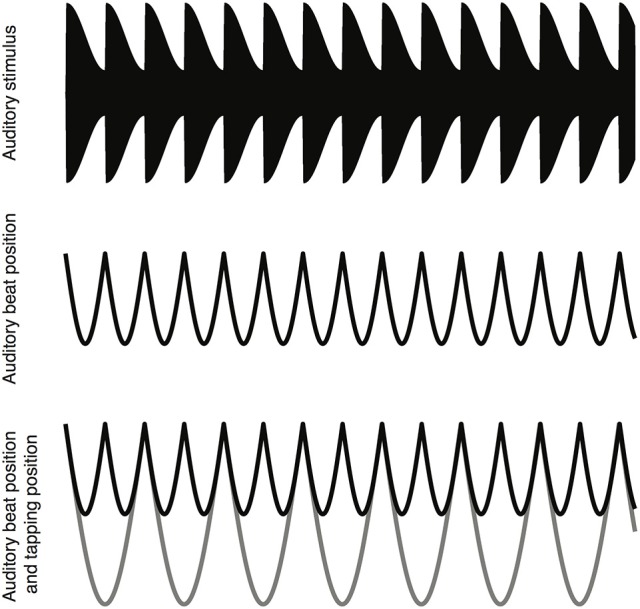
A schematic depiction of the task. A six second sample of the auditory stimulus waveform **(top)**. The location of the auditory beats **(middle)**. The location of the auditory beats (black line) and the location of the finger taps (gray line) **(bottom)**.

The auditory beat stimuli used in the present study were adapted from those used by Nozaradan et al. (2015) and are shown schematically in Figure 1. The stimulus consisted of an amplitude modulated pure tone. The pure tone had a frequency of 333.33 Hz and a duration of 33 s. To introduce the auditory beat the pure tone was amplitude modulated using an asymmetric Hann window with a 12 ms rise and a 404 ms fall time, giving a beat of 2.4 Hz. The modulation depth was 0.25 of the peak amplitude. The beat frequency of 2.4 Hz was of interest because previous studies have shown differences between dyslexic and typically-developing controls in oscillatory brain activity within this range (Power et al., 2013), and because Nozaradan et al. (2015) demonstrated that this beat frequency produces a measurable beat-related SS-EP in EEG. The auditory stimuli were generated using Matlab 2014b (The MathWork) and exported to wave files (16 bit, sample rate 10 kHZ), before being presented using NBS Presentation (Neurobehavioral Systems). The auditory stimuli were delivered binaurally through foam-tipped insert ear-phones (ER-1, Etymotic Research) at a comfortable hearing level. See Figure 1 for a schematic of the stimulus waveform and the timing of the auditory beats.

### Tests of reading, non-word reading, spelling, phonology, auditory discrimination, and IQ

Four subscales from the WISC (Wechsler Intelligence Scales for Children; Wechsler, 1992) were used as a basis for group matching in the wider cohort. A full-scale IQ (FSIQ) was pro-rated from two verbal (Vocabulary, Similarities) and two non-verbal (Picture Arrangement, Block Design) subscales following Sattler (1982). As shown in Table 1, the EEG groups did not differ in FSIQ, but did differ in their reading and spelling performance. Two standardized tests of ability were administered [the British Ability Scale (BAS) single word reading and spelling subscales, Elliott et al., 1996 and the Test of Word Reading Efficiency (TOWRE) word and non-word subscales, Torgesen et al., 1999]. The children also received a standardized measure of rhyme awareness from the Phonological Assessment Battery (PhAB, Frederickson and Frith, 1997) and experimental psychoacoustic measures of auditory rise time and duration thresholds (described below). The group data are shown in Table 1.

### Psychoacoustic tasks for rise time and duration

The auditory tasks used a child-friendly AXB or 2IFC “Dinosaur” threshold estimation program, originally created by Dorothy Bishop (Oxford University), and adapted for a previous study by Martina Huss, University of Cambridge (see Goswami et al., 2013). The psychoacoustic stimuli were presented binaurally through headphones at 75 dB SPL. Earphone sensitivity was calculated using a Zwislocki coupler in one ear of a KEMAR manikin (Burkhard and Sachs, 1975). Children's responses were recorded on the keyboard by the experimenter. The amended Dinosaur programme used an adaptive staircase procedure (Levitt, 1971) with a combined 2-down 1-up and 3-down 1-up procedure; after two reversals, the 2-down 1-up staircase procedure changes into 3-down 1-up. The step size halves after the fourth and sixth reversal. A test run typically terminates after eight response reversals or alternatively after the maximum possible 40 trials. The threshold score was calculated using the mean of the last four reversals.

#### Amplitude envelope onset (rise time) task (1 rise)

A rise time discrimination task in AXB format, the “1 Rise” task from our previous studies (e.g., Goswami et al., 2013), was given on two separate occasions to the participating children. Three 800 ms tones were presented on each trial, with 500 ms ISIs. Two (standard) tones had a 15 ms linear rise time envelope, 735 ms steady state, and a 50 ms linear fall time. The third tone varied the linear onset rise time with the longest rise time being 300 ms. Children were introduced to three cartoon dinosaurs. It was explained that each dinosaur would make a sound and that the child's task was to decide which dinosaur's sound was different from the other two and had a softer rising sound (longer rise time). The child then participated in five practice trials. Feedback was given after every trial by the computer software. During the practice period this was accompanied by further verbal explanation and reinforcement by the researcher.

#### Duration task

This was a duration discrimination task in AXB format. Three tones were presented on each trial, with 500 ms ISIs. The standard was a pure tone with a duration of 125 ms and a frequency of 500 Hz, presented at 75 dB SPL. The duration of the third tone ranged logarithmically from 125 to 250 ms. The children were introduced to three cartoon animals (mice). It was explained that each would make a sound, and the child's job was to decide whose sound was longer. The task was given once.

### Neural and behavioral entrainment paradigm

To examine neural and behavioral entrainment to auditory stimuli, participants listened to the auditory beat in three conditions while EEG was recorded: two tapping conditions, and a listening-only condition. In the listening-only condition, participants were instructed to sit still and listen to the auditory stimulus. They were informed that they would sometimes hear a brief break, or gap, in the sound. At the end of each trial, they were asked to indicate whether the preceding stimulus contained a gap, and they were given feedback about whether their response was correct. In order to create the gap, a 250 ms silent period was added to the stimulus at a random location. Two out of the eight stimuli in the block contained a silent period, and these trials were dropped from the analysis. In the tapping conditions, participants were instructed to tap along to every second beat of the auditory stimulus (1.2 Hz). This was done with both the right hand (Right hand tapping condition) and the left hand (Left hand tapping condition). Figure 1C shows the timing of the taps in relation to the stimulus beats. A period of practice was given prior to the beginning of EEG recording. To record the timing of the taps, the participant tapped on the spacebar of a computer keyboard by performing small up and down movements with the fingers. While the tapping of the spacebar did produce a small amount of auditory feedback, the magnitude of this feedback was reduced by the use of insert ear-phones with foam tips. Participants were instructed to begin their tapping as soon as possible and to try and maintain a steady pace. Each participant performed eight trials per block, with each condition type repeating three times, giving a total of nine blocks.

### EEG recording and analysis

Electroencephalogram (EEG) recordings were performed with a 128-Channel electrode net (Electrical Geodesics Inc.) using a common vertex (Cz) reference. Data was re-referenced to the average reference prior to analysis. Raw EEG was recorded at a 500 Hz sampling rate inside an electrically shielded room, with electrode impedance kept below 50 kΩ. Pre-processing of the EEG data followed the procedure outlined in Nozaradan et al. (2015). The continuous EEG data were filtered using a 0.1 Hz high-pass filter to remove slow drifts, and notch filtered with corner frequencies of 49 and 51 Hz, to remove line noise. The continuous data were then segmented into 32 s long epoch from +1 to +33 s after stimulus onset to remove any transient auditory evoked potentials. Ocular artifacts were removed using Independent Component Analysis. These pre-processing steps were performed in EEGLAB (Delorme and Makeig, 2004). The EEG epochs were then averaged by subject and condition. Averaging the time domain ensures that only frequency components that are phase-locked to the stimulus onset will be present in the subsequent analyses.

#### Time domain analysis

In order to examine potential group differences in the phase of entrainment to the 2.4 Hz beat, condition averages (Auditory only, Right hand tapping, Left hand tapping) were first bandpass filtered using a 12th order Chebyshev filter, with corner frequencies of 2 and 2.8 Hz (65 dB stopband attenuation). Furthermore, following Nozaradan et al. (2015), data analyses were restricted to a subset of frontal electrodes that contained the most power in the 2.4 Hz band (here electrodes FCz, FC1, FC4, AFF1, F1h, F2h, FFC6h, FCC1h, FCC4h, AFp3, AFz, AFF4h; 10-5 positions).

To examine phase difference, the entire epoch was split into 416 ms partially overlapping time bins. This time window size corresponds to exactly two beats (or one tap). To create subsequent bins after the first, the time window was advanced 104 ms, or exactly half a beat. In order to examine the phase difference between the two groups, a 2.4 Hz sinusoid was generated and binned in the same way as the EEG signal. We then calculated the phase difference, in each bin, between the sinusoid and the EEG signal for the DYS group and the CA group, respectively. Phase differences were calculated by determining the time lag between the two signals using a cross-correlation. This time lag was then converted into a phase angle. The phase difference in each of the time bins could then be used to calculate the average phase difference between the EEG signal and the 2.4 Hz sinusoid for each participant. These values were analyzed using the Watson-Williams test (implemented in the Matlab CricStats toolbox).

#### Frequency domain analysis

EEG data were transformed into the frequency domain using Matlab's (The MathWorks Inc.) built in fast Fourier transform algorithm. This results in a frequency domain representation with a resolution of approximately 0.031 Hz. The resulting frequency spectra were then subjected to the noise cancelation procedure outlined in Nozaradan et al. (2015). This noise cancelation procedure involves subtracting, for each frequency bin, the average amplitude from the neighboring frequency bins ranging from −0.15 to −0.09 and from +0.09 to +0.15 Hz. From the noise canceled spectra, the amplitude of a 1.2, 2.4, and 3.6 Hz SS-EP can be calculated as the mean amplitude between 1.160 and 1.221, 2.380 and 2.441, and 3.571 and 3.632 Hz, respectively. For the primary frequency domain analysis, we followed Nozaradan et al. (2015), and estimated the amplitude of each SS-EP over the entire scalp array thereby avoiding any electrode selection bias. The presence of an SS-EP in each condition was determined using one sample *t*-tests. These were conducted on the entire sample of children as well as on each group separately, to ensure that the paradigm worked as expected. Subsequent group comparisons were performed on SS-EP amplitude within each condition using Welch's *t*-test. As differences in the amplitude between conditions and between frequency bands were not of interest, and to avoid unnecessarily inflating our Type-I error rate, we omitted any between condition comparisons either within groups or between groups—that is, we did not conduct any ANOVAs with condition or frequency as factors.

## Results

### Behavioral tapping data

The tapping data were analyzed using circular statistics. Circular statistics produce two metrics, mean direction (equivalent to mean phase angle), and vector length. Mean direction corresponds to how accurately the participant was able to align their tapping output with the beat, while vector length provides a measure of how consistently the participant was able to space their taps. Left handed tapping and right handed tapping were analyzed separately. A Watson-Williams test was used to analyse mean direction. For vector length, however, the data were first logit transformed and then analyzed using two sample *t*-tests.

For mean direction, there was no significant difference between the CA group and the DYS group for the Left hand tapping condition, *F*_(1, 70)_ = 0.001, *p* = 0.973, nor for the Right hand tapping condition, *F*_(1, 22)_ = 0.256, *p* = 0.618. For vector length, *t*-tests revealed no significant group difference for either the Left hand tapping condition, *t*_(16.935)_ = −1.698, *p* = 0.108, or the Right hand tapping condition, *t*_(21.462)_ = −2.009, *p* = 0.057. Accordingly, as no significant behavioral differences were found between the two groups, any differences that may be found in the EEG are unlikely to be explained by group differences in tapping behavior.

### EEG data

In line with our previous work (Power et al., 2013), we first explored whether the phase of entrainment would differ between children with dyslexia and control children for the 2.4 Hz stimulation. As the external beat was at 2.4 Hz, this analysis enabled us to assess whether neural phase alignment (mean phase angle) for the two groups differed in accuracy both during passive listening (Auditory only condition) and when tapping to the beat (Right hand tapping, Left hand tapping). We then computed the SS-EPs following the frequency domain methods (see Section Materials and Methods), to see whether there would be group differences in the SS-EPs at the different frequencies of interest (1.2, 2.4, 3.6 Hz).

### Time domain results

#### 2.4 Hz beat entrainment

**Figure 2:**
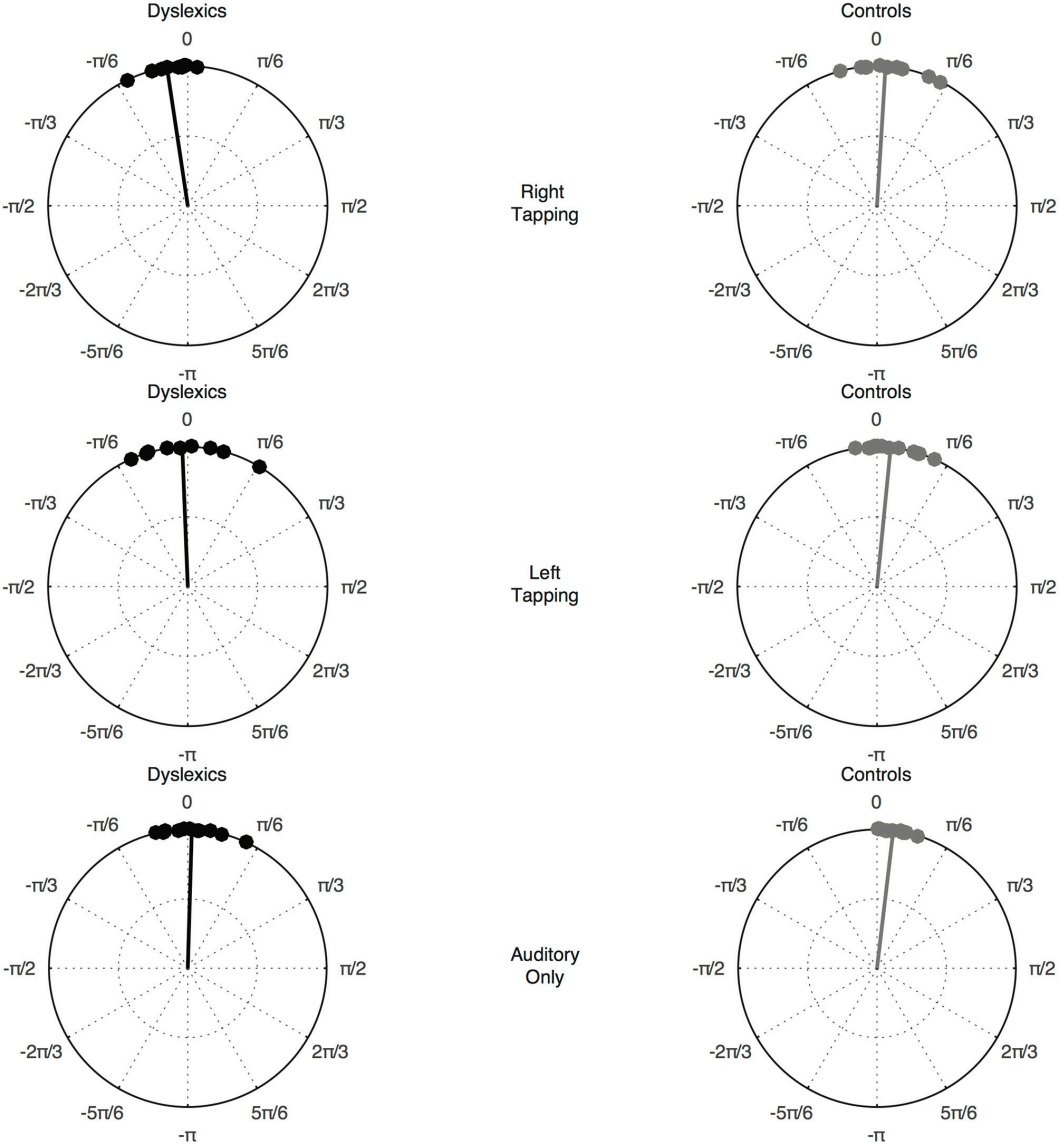
Preferred phase angle by group and condition. The top pair of circular plots shows the Right hand tapping condition, the middle pair of circular plots shows the Left hand tapping condition, and the bottom pair of circular plots shows the Auditory only condition. Preferred phase angle in each case is depicted by the solid line.

The Watson-Williams test revealed a significant difference in phase between the DYS and CA group—that is, the phase difference between the EEG signal and the 2.4 Hz sinusoid was different between the DYS and CA group—*F*_(1, 22)_ = 7.039, *p* = 0.015. Data for each group by condition is presented in Figure 2.

#### Group differences in phase consistency

To explore potential differences by group in phase consistency, we ran a 2 × 3 (Group × Condition) ANOVA, taking the logit transformed vector length as the dependent variable. We found no difference in phase consistency between the CA group and the DYS group, *F*_(1, 22)_ = 1.582, *p* = 0.222, $\eta_{\text{G}}^{2}$ = 0.039. Furthermore, there was no significant main effect of Condition, *F*_(4, 88)_ = 1.485, *p* = 0.233, $\eta_{\text{G}}^{2}$ = 0.028, ϵ = 0.629, and no significant Group × Condition interaction, *F*_(4, 88)_ = 0.601, *p* = 0.589, $\eta_{\text{G}}^{2}$ = 0.012, ϵ = 0.629. This suggests that both groups showed equivalent strength of entrainment. Equivalent strength of entrainment for children with dyslexia and controls was also reported by Power et al. (2013) in the rhythmic speech perception task.

In the current study, group differences hence emerged only when preferred phase was considered, suggestive of phase synchronization to less informative temporal information by children with developmental dyslexia. Indeed, inspection of Figure 2 suggests that they were synchronizing ahead of the beat—their preferred phase was too early, consistent with our prior dyslexia studies using non-motor paradigms (see Goswami et al., 2017, for a review).

### Frequency domain results

**Figure 3:**
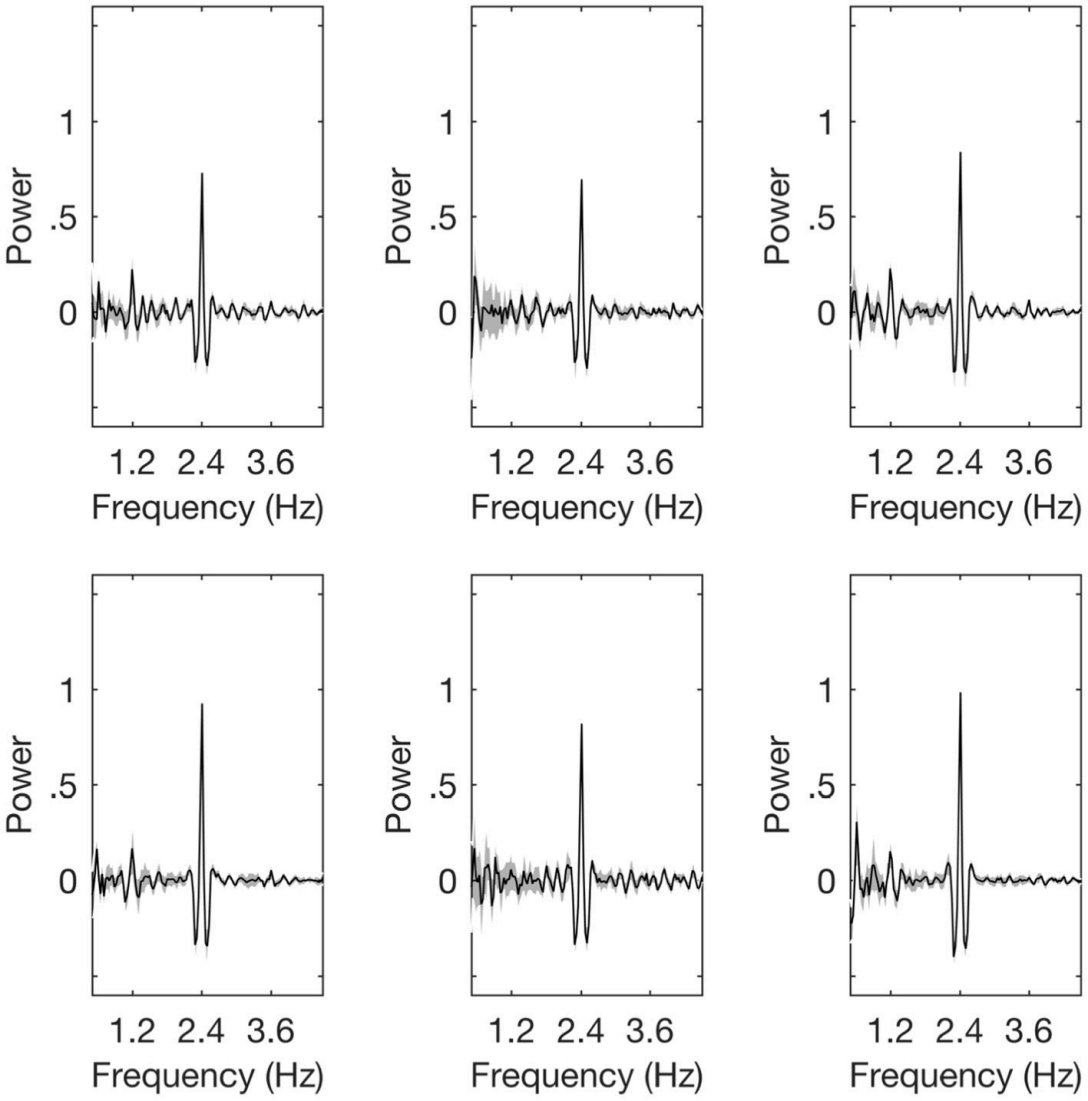
Spectral plots. The plots are, respectively, for the control children (top row) and the children with dyslexia (bottom row). The left hand panels show the Left hand tapping condition, the central panels show the Auditory only condition, and the right panels show the Right hand tapping condition.

The magnitude of the SS-EPs were determined by calculating the average amplitude in the frequency domain signal for frequency bins centered at either 1.2, 2.4, or 3.6 Hz (see Section Materials and Methods). To remove any bias due to electrode selection, all initial frequency analyses were performed on the entire electrode array. However, following Nozaradan et al. (2015), a follow-up exploratory analysis was conducted on the 2.4 Hz SS-EP where we restricted data analyses to a subset of frontal electrodes that contained the most power in the 2.4 Hz band (electrodes Fez, FC1, FC4, AFF1, F1h, F2h, FFC6h, FCC1h, FCC4h, AFp3, AFz, AFF4h; 10-5 positions). The spectral plots for each of the three conditions is shown in Figure 3.

#### Movement-related 1.2 Hz SS-EP

Following Nozaradan et al. (2015), we first checked for the presence of a 1.2 Hz movement-related SS-EP. For the full sample, our results replicated the findings of Nozaradan et al. (2015), and we found a significant SS-EP in the Right hand tapping condition, *t*_(23)_ = 5.700, *p* < 0.001, and the Left hand tapping condition, *t*_(23)_ = 3.666, *p* = 0.001, but not the Auditory only condition, *t*_(23)_ = 1.383, *p* = 0.180. This is expected, as there should be no movement-related SS-EP during passive listening to a 2.4 Hz beat.

When examining each of the groups separately, we again replicated the results of Nozaradan et al. (2015). For the CA group, we found a significant SS-EP in the Right hand tapping condition, *t*_(12)_ = 4.282, *p* = 0.001, and the Left hand tapping condition, *t*_(12)_ = 2.718, *p* = 0.019, but not in the Auditory only condition, *t*_(12)_ = 1.223, *p* = 0.245. And for the DYS group, we again found a significant SS-EP in the Right hand tapping condition, *t*_(10)_ = 3.806, *p* = 0.003, and the Left hand tapping condition, *t*_(10)_ = 2.347, *p* = 0.041, but not the Auditory only condition, *t*_(10)_ = 0.716, *p* = 0.491.

Taken together, these results replicate the findings presented by Nozaradan et al. (2015), and suggest that the paradigm worked as expected in our child sample.

##### Group differences

**Figure 4:**
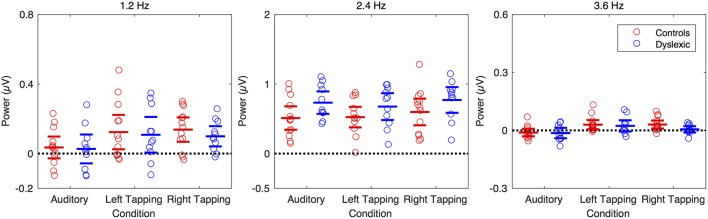
Amplitude of the SS-EPs by group and condition for a sub-set of the electrodes. The left hand panel shows the 1.2 Hz band, the middle panel shows the 2.4 Hz band, and the right panel shows the 3.6 Hz band. Children with dyslexia are in blue and control children are in red. The electrodes were FFCz, FC1, FC4, AFF1, F1h, F2h, FFC6h, FCC1h, FCC4h, AFp3, AFz, AFF4h for the 2.5 Hz band while all electrodes were used for the 1.2 Hz and 3.6 Hz bands.

Turning our attention to possible group differences, we examined whether the magnitude of any of the observed movement-related SS-EPs differed between the two groups. We found no group differences for the Right hand tapping condition, *t*_(21.7)_ = 0.93, *p* = 0.363, the Left hand tapping condition, *t*_(21.76)_ = 0.237, *p* = 0.815, or the Auditory only condition *t*_(19.64)_ = 0.18, *p* = 0.859. These data are shown in Figure 4 (left panel).

#### Beat-related 2.4 Hz SS-EP

We next checked for the presence of a 2.4 Hz beat-related SS-EP. For the full sample, our results again replicated the findings of Nozaradan et al. (2015). We observed a significant SS-EP in the Right hand tapping condition, *t*_(23)_ = 11.423, *p* < 0.001, the Left hand tapping condition, *t*_(23)_ = 10.760, *p* < 0.001, and the Auditory only condition, *t*_(23)_ = 10.097, *p* < 0.001. Restricting our analysis to each group separately, we again replicated the results of Nozaradan et al. (2015) finding a significant beat-related SS-EP in all three conditions for the CA children [Right hand tapping, *t*_(12)_ = 6.745, *p* < 0.001; Left hand tapping, *t*_(12)_ = 7.248, *p* < 0.001; and Auditory only, *t*_(12)_ = 6.146, *p* < 0.001], and in all three conditions for the DYS children [Right hand tapping, *t*_(10)_ = 10.714, *p* < 0.001; Left hand tapping, *t*_(10)_ = 8.336, *p* < 0.001; and Auditory only, *t*_(10)_ = 8.703, *p* < 0.001].

##### Group differences

Of primary interest was whether there were any differences in the magnitude of observed SS-EPs between the groups. As with the 1.2 Hz movement-related SS-EP, we observed no differences in SS-EP magnitude between the two groups for the Right hand tapping condition, *t*_(21.19)_ = −0.995, *p* = 0.331, the Left hand tapping condition, *t*_(21.07)_ = −1.423, *p* = 0.169, or the Auditory only condition, *t*_(21.82)_ = −0.846, *p* = 0.407.

As this was unexpected given the prior dyslexia literature, and to improve the signal to noise ratio (in line with the time-domain analysis reported by Nozaradan et al., 2015), we conducted a further exploratory analysis with a subset of electrodes corresponding with the maximum 2.4 Hz response. Even when the group comparison was restricted to this subset of electrodes, we found no significant difference in the magnitude of the beat-related SS-EP for either of the tapping conditions [Left hand tapping, *t*_(19.78)_ = −1.379, *p* = 0.183; Right hand tapping, *t*_(21.98)_ = −1.428, *p* = 0.167]. For the Auditory only condition, however, we found a significant difference in the magnitude of the beat-related SS-EP, *t*_(21.99)_ = −2.088, *p* = 0.049, with the beat-related SS-EP being significantly larger in the DYS group (*M* = 0.732) relative to the CA Group (*M* = 0.511, Δ*M* = 0.221, 95% CI[0.001, 0.441]). To quantify the evidential weight for the increased amplitude in the DYS group relative to the CA group, we conducted a Bayes factor analysis (Morey and Rouder, 2011) comparing an alternative hypothesis, that the effect is positive, with a null hypothesis, that the effect is zero. The resulting Bayes factor can be interpreted as an odds-ratio. The results suggest that it is approximately three times more likely that the difference is positive relative to the difference being zero (JZS BF_+0_ = 3.102). Thus, even though the evidence is weak, there is a suggestion that passive auditory processing of the beat differed between groups. This would need to be explored further in a higher-powered study. These data are shown in Figure 4 (middle panel).

#### Cross-modulation 3.6 Hz SS-EP

Once again, we followed Nozaradan et al. (2015) and checked whether the cross-modulation SS-EP was present in the two tapping conditions and absent in the Auditory only condition. For the full sample, we were able to replicate their results, finding a significant SS-EP in the Right hand tapping condition, *t*_(23)_ = 2.889, *p* = 0.008, and the Left hand tapping condition, *t*_(23)_ = 3.254, *p* = 0.003, but not in the Auditory only condition, *t*_(23)_ = −1.721, *p* = 0.099. When restricting our analysis to each group separately, however, some group differences emerged. The typically-developing CA group showed the expected pattern, with a significant SS-EP in the Right hand tapping condition, *t*_(12)_ = 3.170, *p* = 0.008, the Left hand tapping condition, *t*_(12)_ = 2.720, *p* = 0.019, but not the Auditory only condition, *t*_(12)_ = −1.260, *p* = 0.232. However, for the DYS group, no significant SS-EPs were observed in any of the conditions. No significant SS-EP was found in the Right hand tapping condition, *t*_(10)_ = 0.759, *p* = 0.465, nor the Left hand tapping condition, *t*_(10)_ = 1.800, *p* = 0.102, nor the Auditory only condition, *t*_(10)_ = −1.134, *p* = 0.283. Taken together, the data for the 3.6 Hz SS-EPs suggest that only the typically-developing children displayed the pattern of results found by Nozaradan et al. (2015), while the children with dyslexia did not. This could imply that sensorimotor integration is impaired in developmental dyslexia. However, because a difference in significance does not guarantee a significant group difference, we next examined the group difference directly.

##### Group differences

We compared the two groups directly to determine whether there were any statistically significant differences in the magnitude of observed SS-EPs. This analysis revealed no differences in SS-EP magnitude between the two groups for the Right hand tapping condition, *t*_(21.33)_ = 2.065, *p* = 0.051, the Left hand tapping condition, *t*_(20.47)_ = 0.357, *p* = 0.724, or the Auditory only condition, *t*_(19.15)_ = 0.163, *p* = 0.873. Statistically, therefore, while no 3.6 Hz SS-EPs were observed in the DYS group, the measured power amplitudes in the 3.6 Hz range did not differ between the two groups. This is wholly unsurprising, in that the first analysis relies only on the within-group variance, which is expected to be less than the between-group variance on which the second analysis relies. However, the difference between the CA group and the DYS group for the right hand tapping condition was approaching significance (*p* = 0.051). To quantify the evidential weight for the increased amplitude in the CA group relative to the DYS group, we conducted a Bayes factor analysis for a positive effect compared with a null hypothesis effect. The results suggest that it is approximately three times more likely that the difference is positive relative to the difference being zero (JZS BF_+0_ = 2.862). Thus, while the evidence is weak, these results may suggest that the internal representation of the beat was impaired for the children with dyslexia when tapping with the preferred (right) hand. Sensorimotor integration in dyslexia is therefore possibly weaker. However, this possibility would need to be explored further in a higher-powered study. These data are shown in Figure 4 (right panel).

### Brain-behavior correlations

To examine whether the neural phase differences (time domain) were related to children's progress in learning to read, we computed circular-linear correlations between preferred phase angle in the three conditions and the behavioral measures administered to our sample of children (see Table 2). For completeness, we also computed correlations for all the different SS-EP measures (frequency domain). However, most correlations in the frequency domain were non-significant, the exceptions occurring for the 2.4 Hz beat-related SS-EPs. These latter data are also shown in Table 2. As would be predicted by both TS theory and DAT, the associations with reading/phonology that reached significance were for neural phase synchronization during both auditory-only stimulation and right hand tapping. Individual differences in auditory discrimination of both rise time and duration were also related to neural phase synchronization for both tapping and passive listening, in line with TS theory. Regarding 2.4 Hz SS-EP power in the Auditory only condition, individual differences in neural power were significantly related to all the measures of reading. The negative correlations indicate that greater power is associated with weaker reading development. Thus, there is evidence that the accuracy of neural synchronization to an external beat is related to progress in reading and phonological development, both when measured via passive entrainment to the beat (Auditory only condition) and when sensorimotor integration is required (Right hand tapping).

**Table 2:** Correlations between phase and power at 2.4 Hz and behavioral measures, for the subset of electrodes.

	**Right tap phase**	**Left tap phase**	**Auditory only phase**	**Right tap SS-EP power**	**Left tap SS-EP power**	**Auditory only SS-EP power**
Reading age in months	0.51^*^	0.26	0.49^+^	−0.11	−0.07	0.40^+^
BAS reading SS	0.43	0.21	0.38	−0.19	−0.19	−0.45^*^
BAS spelling SS	0.41	0.25	0.34	−0.11	−0.17	−0.32
TOWRE total SS	0.31	0.17	0.35	−0.18	−0.19	−0.42^*^
PhAB rhyme SS	0.52^*^	0.28	0.41	−0.10	−0.14	−0.27
PhAB rhyme #trials correct	0.45^+^	0.30	0.51^*^	−0.16	−0.23	−0.31
Rise time	0.39	0.23	0.53^*^	0.02	0.08	0.17
Duration	0.53^*^	0.17	0.42	0.01	0	0.22

* *p < 0.05*,

+ *p < 0.06*.

*BAS, British Ability Scales, SS, standard score, TOWRE, Test of Word Reading Efficiency, PhAB, Phonological Assessment Battery*.

## Discussion

Here, we set out to investigate the neural mechanisms that may underpin the developmental relationships between the precision of beat synchronization, children's phonological awareness, and their progress in reading (Thomson and Goswami, 2008; Corriveau and Goswami, 2009; Dellatolas et al., 2009; Flaugnacco et al., 2014; Woodruff Carr et al., 2014). Precise specification of neural mechanisms should enable the optimization of remedial programmes based on improving temporal synchronization in children, for example programmes based on drumming and other forms of rhythm production, which have been shown to enhance both children's phonological awareness and their reading development (Overy et al., 2003; Degé and Schwarzer, 2011; Bhide et al., 2013; Slater et al., 2014; Flaugnacco et al., 2015; Serrallach et al., 2016). Following prior work by Nozaradan et al. (2015), we aimed to use beat-related SS-EPs to disentangle sensory- and motor-related neural beat entrainment in children with developmental dyslexia and age-matched control children. Consistent with the prior dyslexia literature on neural entrainment, which has revealed atypical entrainment to both speech and non-speech rhythmic stimuli in the delta band (~1–3 Hz, see Hämäläinen et al., 2012; Power et al., 2013, 2016; Soltész et al., 2013; Molinaro et al., 2016), we also analyzed neural phase alignment (preferred phase angle) and phase consistency for the 2.4 Hz rhythmic stimulus.

The data showed atypical neural beat-driven entrainment in dyslexia in terms of preferred phase angle, and greater neural power when passively tracking the beat (2.4 Hz SS-EP in the Auditory only condition, *p* < 0.05, BF_+0_ = 3.102). The data were suggestive of an impaired internal representation of the beat during sensorimotor integration during right handed tapping (SS-EP at 3.6 Hz, *p* = 0.051, BF_+0_ = 2.862). There were no group differences for motor-related neural activity alone (1.2 Hz SS-EPs). As all children were right handed and still relatively young, it is likely that the variability of responding with the left hand precluded any group differences for the Left hand tapping condition (in their study with neurotypical adults, Nozaradan et al. (2015) also found significant effects for Left hand tapping). Both the neural time domain (preferred phase) and frequency domain (SS-EP at 2.4 Hz, Auditory only condition) measures showed significant correlations with the development of single word reading and phonological awareness.

Overall, our neural data support the view that there is an auditory rhythm perception deficit in developmental dyslexia, and that this sensory deficit affects the temporal precision of action. Given the results obtained in temporal remedial programmes based on practicing beat synchronization (see Degé and Schwarzer, 2011; Bhide et al., 2013; Slater et al., 2014; Flaugnacco et al., 2015; Serrallach et al., 2016), these auditory sensory deficits do not seem to prevent children from benefitting from rhythmic practice in the motor modality. However, the current data leave open the theoretical question of whether such behavioral programmes are beneficial because they improve a primary sensory deficit in auditory rhythm perception in dyslexia via cross-domain sensorimotor coupling (TS theory; Goswami, 2011), or because they improve sensorimotor temporal prediction (DAT theory; Jones, 2008), or both. The likely conclusion is that both theoretical frameworks are related to children's synchronization behavior, because both attention and language processing are supported by oscillatory neural processes.

Regarding TS theory, it has been argued that rhythmic remediation for dyslexia may improve phonological and reading skills by “entraining the oscillators,” as discussed by Goswami and Szucs (2011). This general proposal is also consistent with DAT theory. Goswami and Szucs (2011) proposed that both rhythmic *linguistic* interventions requiring motor production of speech, such as programmes based on singing, oral learning of nursery rhymes and reciting metrical poetry, and rhythmic interventions involving rhythmic motor practice such as beat synchronization, might support the accuracy of entrainment of lower frequency oscillatory networks (theta- and delta-rate networks) in primary auditory cortex, those important for accurate rhythm perception (see Giraud and Poeppel, 2012; Patel, 2012). We have also argued from behavioral data in a musical task based on a 2 Hz pulse (Huss et al., 2011) that the dyslexic brain may not be able to set up a reliable internal acoustic representation of a 2 Hz beat. A 2 Hz beat may represent an approximate periodic structure in language (based on stressed syllables, see Goswami et al., 2013) to which the infant brain may entrain across languages, facilitating language acquisition. Sensitivity to speech rhythm is considered a cross-language precursor of language acquisition (e.g., Mehler et al., 1988). If sensitivity to linguistic beat-based temporal structure is impaired in developmental dyslexia, this would lead to phonological impairments. By TS theory therefore, beneficial effects of rhythmic tapping on phonological development and reading would occur via improved temporal precision of auditory oscillatory networks via coupling with motor oscillators (see also Putkinen et al., 2013; Cason et al., 2015).

Meanwhile, DAT expects that any linguistic improvements that follow rhythmic practice are mediated by improved temporal prediction in the attention system. For language development, improving temporal prediction would improve expectations concerning the location of the most informative portions of the speech signal, such as stressed syllables. As discussed by Kotz and Schwartze (2010), speech perception is inherently linked to rhythmic timing via the perception of rhythmically prominent (stressed) syllables. The patterning of the stressed syllables provides a temporal structure that can be used by the brain in encoding and understanding speech, with these quasi-rhythmic events guiding auditory attention to important information. Indeed, it is known that beat-based and musical primes can improve children's *syntactic* processing as well as their phonological processing (Przybylski et al., 2013; Gordon et al., 2015). The most conservative conclusion from our data is that both auditory oscillators and any other oscillators involved in temporal prediction benefit from practice in beat synchronization via cross-modal integration.

It should be noted that a related account of why beat synchronization may enhance children's phonological awareness, but based on more rapid timescales, has been proposed by Tierney and Kraus (2014, 2016), the Precise Auditory Timing Hypothesis (PATH). Tierney and Kraus noted that the development of phonological awareness depends on precise timing perception, particularly at rapid timescales such as the differences in voice onset time or in formant transitions that distinguish consonants like /b/ and /d/. They noted that children with musical training are better able to distinguish such consonants (Strait et al., 2012), and that musical training may support fine-grained temporal discrimination by enhancing auditory-motor timing. The PATH is also consistent with the current data. The main difference in focus between TS theory and the PATH is in the temporal rates proposed to mediate the audio-motor effects on children's phonological development, namely slow (TS theory, which foregrounds syllabic information <10 Hz) vs. faster (the PATH, which foregrounds phonetic information, usually considered >30 Hz). While PATH focuses on rapid timescales, TS theory accords a special explanatory role to delta-band information. The 2.4 Hz beat utilized here falls within the delta band.

Oscillations in the delta band (~1–3 Hz, see Poeppel, 2014) sit at the top of the neural oscillatory hierarchy for speech encoding in auditory cortex (Gross et al., 2013). Amplitude modulations in the delta band play a key role in rhythmic child-directed speech (English nursery rhymes, Leong and Goswami, 2015) and in infant-directed speech (Leong et al., 2017). Adult studies reveal that neural oscillatory networks for speech act as a hierarchy via both phase-phase and phase-power relations, with delta rate oscillators governing the activity of oscillators at faster rates (Gross et al., 2013). This oscillatory hierarchy matches the *acoustic hierarchy* of AMs in English nursery rhymes (Goswami and Leong, 2013). Both auditory and neural studies suggest that oscillations in the delta band are related to the extraction of syllable stress patterns (e.g., Ghitza and Greenberg, 2009; Ghitza et al., 2013; Poeppel, 2014; Leong and Goswami, 2015). Accordingly, slower delta-rate temporal information may be an important target for remedial rhythmic programmes involving young children. One way to explore the temporal rate issue in more depth may be to contrast the effects on phonological awareness of general musical remediation with remediation focused specifically on delta-band rhythms.

In conclusion, the data reported here are supportive of atypical neural beat entrainment by the dyslexic brain in the delta band (~1–3 Hz), with the children with dyslexia showing a significantly different preferred phase both during passive listening and during beat synchronization. Brain-behavior correlations were found between measures of both reading and phonological development and individual differences in both preferred phase and in the power of SS-EPs at the auditory stimulation rate (2.4 Hz). Overall, the data are supportive of an interpretation of developmental difficulties in beat synchronization driven by impaired auditory perception of the beat. However, whether this conclusion would also apply to other developmental disorders of language learning which present with beat synchronization difficulties (stuttering, SLI) and whether it would apply in languages that are syllable-timed rather than stress-timed, requires further investigation.

## Author contributions

LC and UG designed the experiments, performed the analyses, and drafted the manuscript. LC and HN collected the data.

### Conflict of interest statement

The authors declare that the research was conducted in the absence of any commercial or financial relationships that could be construed as a potential conflict of interest.
